# Minimizer ring implantation versus pouch revision for weight regain and functional failure after Roux-en-Y gastric bypass: A retrospective single-centre analysis

**DOI:** 10.1007/s00423-026-04075-6

**Published:** 2026-05-21

**Authors:** Marius Arbogast, Michelle Hauf, Nicola Schaffner, Magdalena Biraima, Christopher Soll

**Affiliations:** 1 Departement for Surgery, Center for Obesity Surgery, GZO Spital Wetzikon, Wetzikon, Switzerland; 2 Paracelsus Medical University, Salzburg, Austria

**Keywords:** Roux-en-Y gastric bypass, Revisional bariatric surgery, Minimizer ring, Pouch revision, Weight regain after bariatric surgery, Ring-augmented gastric bypass

## Abstract

**Purpose:**

To compare short- to mid-term weight loss outcomes, safety, and functional tolerance between Minimizer ring implantation and pouch revisional surgery in patients presenting with recurrent weight gain or functional failure after Roux-en-Y gastric bypass (RYGB).

**Methods:**

This retrospective observational study included patients undergoing revisional bariatric surgery after RYGB between 2020 and 2025 at a single Swiss bariatric center. Revisional strategies comprised Minimizer ring implantation and pouch-based revisions. Outcomes assessed included percentage total weight loss (%TWL), body mass index (BMI) changes, postoperative complications, reinterventions, and functional tolerance at 3, 6, and 12 months. Non-parametric statistical methods were applied.

**Results:**

27 patients were analyzed (14 Minimizer ring implantations, 13 pouch revisions). Both strategies achieved progressive weight loss with no significant differences in %TWL at 3, 6 and 12 months. Early postoperative complication rates were similar in both groups. Minimizer ring implantation was associated with a higher rate of late intolerance and secondary interventions leading to ring removal.

**Conclusion:**

Within the available 12-month follow-up, no statistically significant difference in weight loss was detected between Minimizer ring implantation and pouch revisional surgery. Minimizer ring implantation was associated with a higher observed burden of intolerance and secondary interventions in this cohort. Pouch revision may provide anatomical correction without foreign-body-related complications. These findings should be considered hypothesis-generating and suggest that future studies should assess cumulative reintervention burden across revisional strategies.

## Introduction

Roux-en-Y gastric bypass (RYGB) is a widely established bariatric procedure, demonstrating significant short- and medium-term weight loss and metabolic improvement [1, 2]. However, long-term follow-up indicates that a substantial proportion of patients experience significant weight regain, which can diminish the durability of these health benefits. In response, the application of silicone rings such as Minimizer or Fobi rings around the gastric pouch has emerged as a strategy to enhance gastric restriction, reduce pouch or anastomosis dilation, and thus mitigate recurrent weight gain (RWG) [2, 3]. In contrast, pouch revisional surgery is also considered a viable corrective measure for weight regain or anatomical failure after RYGB [4]. These procedures are technically demanding and widely perceived to carry higher complication rates than both primary bariatric surgery and the comparatively simpler and quicker implantation of a Minimizer ring [4]. Importantly, ring implantation and pouch revision should not be regarded as mutually exclusive strategies, as combined approaches, such as pouch revision with Minimizer ring placement, have recently been reported as revisional options for patients with recurrent weight gain or suboptimal clinical response after RYGB [5]. While multiple studies report improved weight outcomes with ring augmentation, this approach also carries risks, including dysphagia, ring migration, and food intolerance [2–5]. These concerns are particularly relevant in the revisional setting, even when procedures are performed in experienced bariatric reference centers. Given the variability in patient anatomy, behavioral compliance, and risk profiles, the correct indication for employing ring augmentation versus pouch revisional surgery is critical to ensure that the benefits of enhanced weight control outweigh the potential for postoperative complications [4]. Despite widespread use, the clinical role of silicone ring augmentation after RYGB remains controversial. Reported outcomes vary considerably in terms of weight durability, functional tolerance, and re-intervention rates, limiting generalizability to routine clinical practice. Moreover, the literature predominantly emphasizes weight-based endpoints, while the cumulative burden of postoperative intolerance, endoscopic procedures, and reoperations remains inconsistently captured. As revisional bariatric surgery is increasingly performed in anatomically altered and higher-risk patients, a more comprehensive evaluation is warranted. In this context, direct comparison between ring implantation and anatomical pouch revision may help refine indication criteria beyond weight loss alone. Systematic reviews of revisional RYGB techniques report heterogeneous outcomes and complication profiles, underscoring the lack of consensus regarding optimal strategy selection and highlighting the need for comparative analyses that extend beyond weight loss alone [6]. In this retrospective clinical analysis, we focused on patients treated with either Minimizer ring implantation or pouch-based revision without ring placement, while acknowledging that combined procedures represent a clinically relevant alternative strategy.

## Methods

**Fig. 1:**
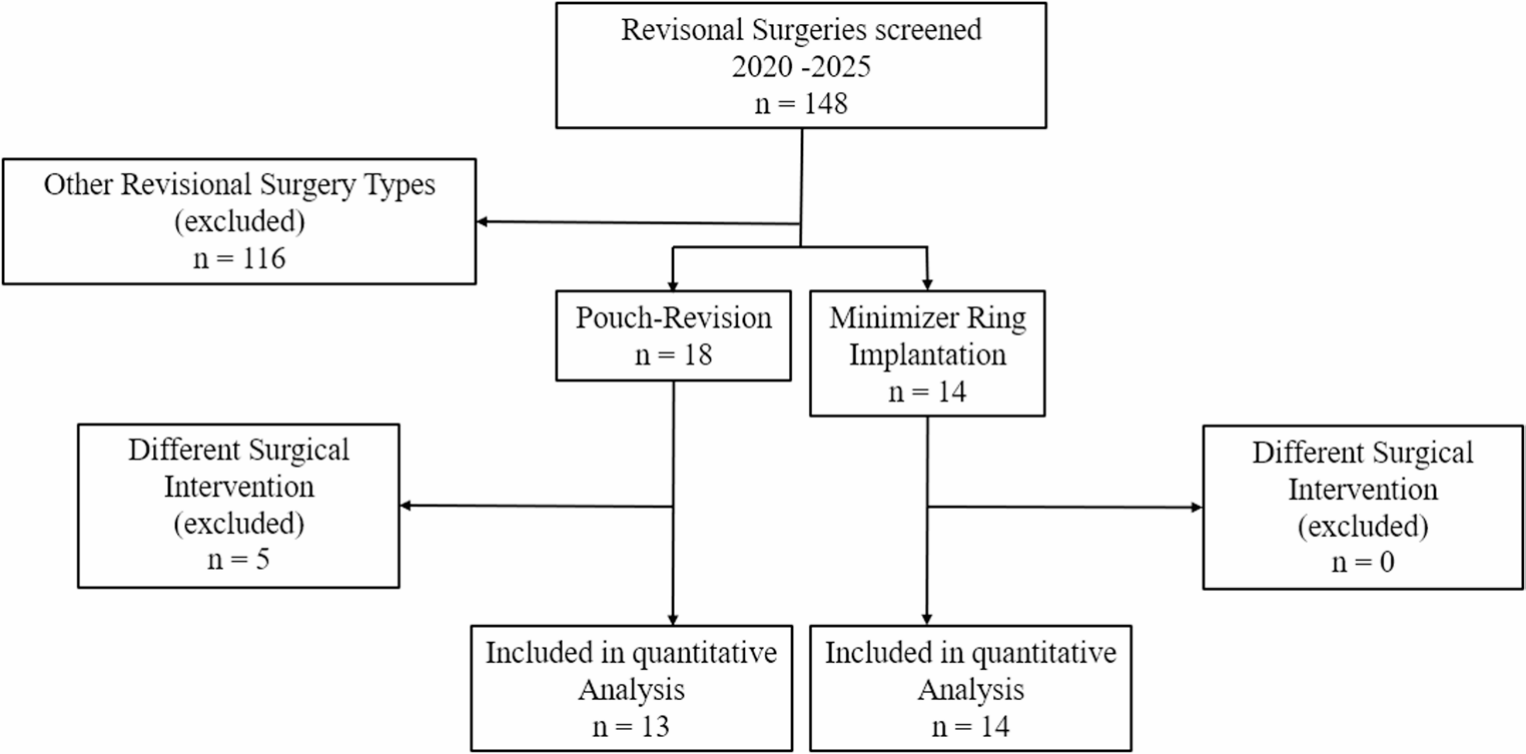
Flow diagram of patient selection and inclusion in the analysis

In this study we evaluated data from revisional bariatric surgeries performed between 2020 and 2025 at our bariatric center in Switzerland. Patients undergoing pouch revision, for restriction failure, due to pouch or anastomotic dilation leading to weight regain and/or therapy resistant dumping, following RYGB were analyzed. Three revision techniques were identified, whereas surgical revisions without ring implantation were pooled into one group. Outcomes assessed included operative duration, postoperative complications hospital stay length, and weight loss at 3, 6 and 12 months. As shown in Fig. 1, a total of 148 revisional bariatric surgeries performed after RYGB were initially screened for inclusion in the analysis. Of these, 116 cases were excluded due to being classified under other types of revisional surgery not relevant to the current comparison. The remaining cases were divided into two intervention groups: 18 pouch revision surgeries and 14 minimizer ring implantations. The pouch revision cohort included patients who underwent full anastomotic revisions, gastric pouch resizing with or without jejunal-crutch resections. Pouch-based procedures were pooled into one group because all were performed with the same therapeutic intent. Although the technical extent of revision differed between pouch resizing, jejunal-crutch resection, and complete gastrojejunal anastomotic revision, all procedures aimed to restore restriction, correct rapid emptying, or address anatomical failure after RYGB. Given the small number of individual procedure types, separate statistical analysis of each pouch-revisional subgroup was not feasible. Within the pouch revisional group, an additional 5 cases were excluded due to differing surgical indications, such as treatment of strictures, marginal ulcers, fistulas or other anatomical findings. The minimizer ring implantation group had no exclusions. After exclusions, both groups were stratified by primary indications for revision, including RWG, loss of restriction or satiety, and postprandial dumping, based on preoperative symptom profiles. Recurrent weight gain or weight recurrence was assessed relative to the individual postoperative nadir and expressed as percentage weight regain from nadir. Owing to the retrospective design, standardized IFSO definitions were not uniformly applied. Revision was indicated by the treating bariatric team based on weight trajectory, symptom recurrence, dietary history and anatomical findings. Restriction failure was defined as loss of satiety or increased meal volume. This was supported where available by radiographic or endoscopic evidence of pouch dilation or a widened gastrojejunal anastomosis. Therapy-resistant dumping was defined as persistent postprandial symptoms despite dietary counselling. These definitions should therefore be interpreted as pragmatic clinical criteria rather than prospectively standardized endpoints. Functional outcomes were assessed retrospectively from clinical follow-up documentation. No validated food tolerance score, dumping severity score or quality-of-life instrument was applied. These outcomes should be interpreted carefully since no standardized patient-reported outcome measures have been obtained. This study was conducted and reported in accordance with the STROBE (Strengthening the Reporting of Observational Studies in Epidemiology) guidelines.

### Statistical analysis

Due to the small sample size and non-normal distribution of continuous variables, non-parametric statistical methods were applied. Continuous variables are reported as medians with interquartile ranges (IQRs), and categorical variables as absolute counts and/or percentages. Comparisons between the Minimizer ring implantation group and the pouch revision group were performed using the Mann-Whitney U test. Categorical variables were compared using Fisher’s exact test or Pearson’s chi-square test, as appropriate. Within group changes in BMI from the time of revision to follow-up at 3, 6, and 12 months were analyzed using the Wilcoxon signed-rank test. Associations between pre-revision percentage weight regain from nadir (%RWG) and postoperative weight loss outcomes (%TWL and %EWL) were assessed using Spearman’s rank correlation coefficient. These correlation analyses were considered exploratory due to the limited sample size. All statistical tests were two-sided, and a p-value < 0.05 was considered statistically significant. Statistical analyses were performed using IBM SPSS Statistics, version 29 (IBM Corp., Armonk, NY, USA).

## Results

### Patient’s characteristics and operative times

A total of 27 patients were included in this analysis, comprising 21 females and 6 males. The gender distribution and demographic characteristics were broadly similar between the two groups. The median age at revision was 41 years (IQR 16), ranging from 27 to 68 years, without a significant difference between the groups (Table 1).

**Table 1:** Comparison of weight-related parameters between the Minimizer ring group and pouch revision group, reported as median and interquartile range (IQR). *P* values were derived using the Mann-Whitney U test

	Group A (Minimizer Ring) Media (IQR)	Group B (Pouch Revision) Media (IQR)	
	*n*=14	*n*=13	*p*-value <0.05
BMI preRYGB (kg/m^2^)	42.1 (37.6 - 46.6)	41.3 (38.5 - 46.7)	0.8462
BMI nadir (kg/m^2^)	27.0 (26.3 - 29.2)	31.3 (29.2 - 32.1)	0.0606
RWG (kg/m^2^)	14.1 (11.3 - 17.3)	10.4 (8.4 - 15.0	0.3826
RWG (%)	21.5 (16.7 - 34.5)	15.9 (4.7 - 20.1)	0.0523

**Fig. 2:**
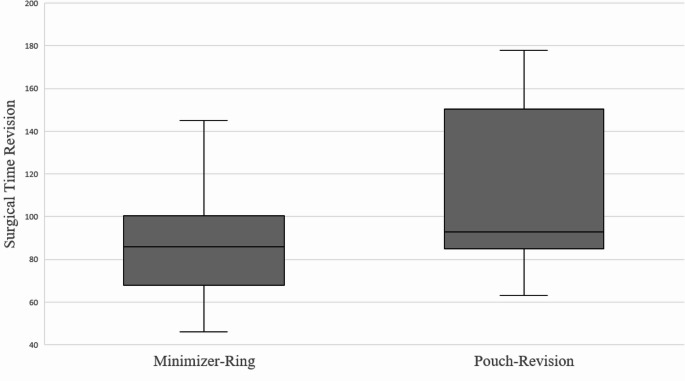
Comparison of operative time between minimizer ring implantation and pouch revision

Prior to the initial RYGB, the median BMI across all patients was 42.1 kg/m², with a broad distribution indicating interindividual variability. There were no significant differences in baseline BMI between the two cohorts. Following primary RYGB, patients achieved an average BMI reduction of -12.45 kg/m². The median time to nadir weight was 535 days (1.47 years) with an interquartile range of 329.5 days (0.90 years). The median nadir BMI was lower in the Minimizer group (27.0 kg/m²) than in the pouch revision group (31.25 kg/m²), with female patients generally reaching slightly lower nadir values than males. Prior to re-evaluation for revision, patients experienced a median BMI increase of 5.3 kg/m², equating to a median 16.4% RWG from nadir. While not statistically significant, the Minimizer group exhibited a greater degree of RWG with data showing a median of 21.48% BMI (14.1 kg/m²) compared to 15.94% (10.42 kg/m²) in the pouch revision group. The numerically higher pre-revisional weight recurrence in the Minimizer group may indicate baseline imbalance and should be considered when interpreting between-group outcome comparisons. Operative time for the primary RYGB procedure had a median duration of 79 min (IQR 50), with no significant difference observed between groups. Revisional procedures had a median operative time of 89 min. The Minimizer group demonstrated shorter and less variable operative times (median 86 min, IQR 26.5) compared to the pouch revision group (median 93 min, IQR 61), indicating greater procedural complexity (Fig. 2). Despite a numerically shorter operative time in the Minimizer group, non-parametric comparison revealed no statistically significant difference between groups (U = 55, *p* > 0.05).

### Preoperative symptoms and postoperative outcomes

In the pouch revision group, 4 patients reported dumping syndrome and 11 reported a loss of restriction post-RYGB. Of the 13 patients assessed, 8 reported increased food portion sizes, and 5 reverted to pre-RYGB portion sizes. Nine patients underwent preoperative gastroscopy, and 12 received radiographic contrast swallow of the esophagus and gastric pouch to assess pouch size, and anastomotic integrity. In the Minimizer Ring group, 7 patients reported post-RYGB dumping syndrome and 13 experienced a loss of restriction(Table 2). All patients reported increased food intake, with 11 noting substantially larger portion sizes. Preoperative gastroscopy was performed in 13 of the 14 patients, and in all patients a radiographic assessment for surgical planning was performed using a contrast swallow study. In patients without documented preoperative gastroscopy, surgical planning was based on contrast swallow imaging or prior endoscopic findings. Missing endoscopic data may reflect incomplete external documentation. Radiographic and endoscopic assessments consistently demonstrated preserved gastrointestinal passage without evidence of obstruction or leakage. However, both modalities frequently revealed a dilated gastric pouch and, more prominently, a widened gastrojejunal anastomosis. Additionally, radiographic studies often showed rapid contrast transit into the jejunum.

**Table 2:** Preoperative system profile and postoperative functional outcomes by revision type

	Pouch Revision (*n*=13)	Minimizer Ring (*n*=14)
Preoperative Symptoms		
Dumping syndrome, *n* (%)	4 (30.8%)	7 (50.0%)
Loss of restriction, *n* (%)	11 (84.6%)	12 (85.7%)
Incresed food portion size, *n* (%)	11 (84.6%)	13 (92.9%)
Postoperative functional outcome		
Improvement in dumping	Majority improved	Variable improvement
Restoration of restriction/ reduced portion size	Sustained in majority	Initially achieved, not sustained in all
Re-intervention affecting function	-	Ring removal (*n*=6)
		Endoscopic removal of impacted food (*n*=1)

Postoperative outcomes differed substantially between groups. In the pouch revision cohort, outcomes were generally favorable, with one major complication requiring re-operation and one case of transient dysphagia managed conservatively. Functional improvement was consistent, with sustained restoration of restriction and improvement in dumping symptoms, and no need for re-intervention. In contrast, the Minimizer ring group showed greater variability. One patient required urgent re-operation due to early ring dislocation with ischemia of the alimentary limb. Additionally, 6 of 14 patients (42.9%) developed ring-related intolerance, including dysphagia, vomiting, and abdominal pain, leading to ring removal. The median time to Minimizer ring removal was 8.36 months (IQR 6.43). While initial restoration of restriction and improvement in dumping symptoms were observed in some patients, these effects were not consistently maintained, and symptom recurrence after ring removal occurred. Overall, pouch revision showed more stable functional outcomes within the available follow-up, whereas Minimizer ring implantation was associated with more device-related complications and variable efficacy (Table 2). Early postoperative complications (Clavien-Dindo) did not differ significantly between groups (*p* = 1.000), nor did documented functional or ring-related intolerance (*p* = 0.816). In contrast, re-operation and endoscopic re-intervention rates were significantly higher in the Minimizer group (χ² = 4.34, *p* = 0.037; Fisher’s exact *p* = 0.048), indicating an increased need for secondary interventions following ring implantation. 

### Weight loss outcomes and correlation analysis

**Fig. 3:**
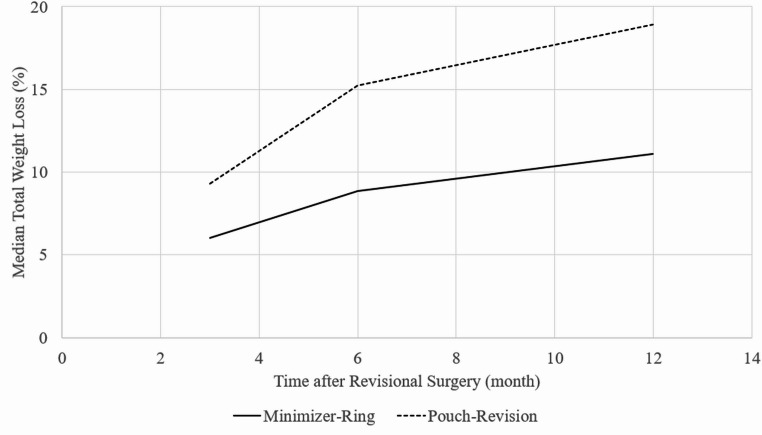
Median total weight loss (%TWL) over time following revisional bariatic surgery, comparing Minimizer ring implantation and pouch revision

Median %TWL increased over time in both treatment groups. At 3 months, median %TWL was numerically higher in the pouch revision group compared with the Minimizer ring group; however, this difference did not reach statistical significance (Mann–Whitney U = 2.0, *p* = 0.068). No statistically significant between-group differences in %TWL were observed at 6 months (U = 34.0, *p* = 0.280) or 12 months (U = 16.0, *p* = 0.302) (Fig. 3). Longitudinally, both cohorts exhibited progressive weight loss at 3, 6, and 12 months. The pouch revision group showed a more pronounced early response, whereas the Minimizer ring group demonstrated a more gradual increase. These results should be interpreted cautiously given the very small sample sizes and the limited availability of follow-up data beyond 6 months, particularly in the Minimizer group due to removal of 42.9% of rings after a median of 8.36 months. These findings indicate that no statistically significant between-group difference in %TWL was detected during short- to mid-term follow-up. Among patients revised primarily for recurrent weight regain, %TWL at 12 months did not differ significantly between treatment strategies (Fisher’s exact test, *p* = 1.000). Within-group analyses demonstrated a significant reduction in median BMI from revision to 6 months in both groups. In the Minimizer ring group, median BMI decreased significantly at 6 months (*p* = 0.019) and remained significantly lower at 12 months (*p* = 0.046). In the pouch revision group, median BMI also decreased significantly at 6 months (*p* = 0.025); however, the reduction at 12 months did not reach statistical significance (*p* = 0.263).

**Fig. 4:**
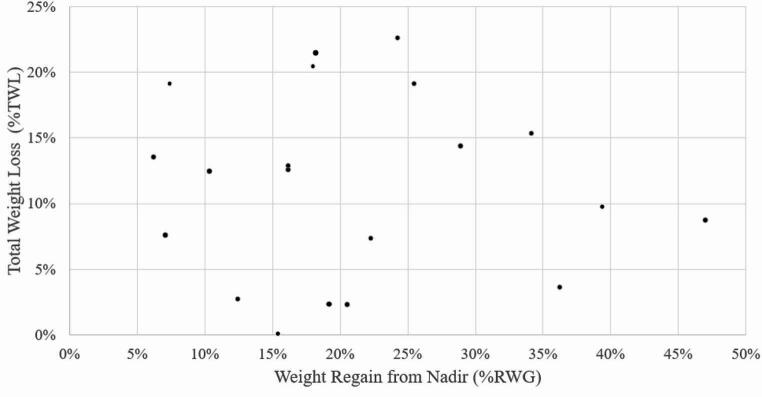
Correlation between percentage weight regain from nadir and percentage total weight loss at 6 months after revisional surgery

To assess whether pre-revisional weight regain influences postoperative outcomes, we analyzed the relationship between %RWG from nadir and %TWL following revisional surgery. No significant association was observed between %RWG and %TWL at 3, 6, or 12 months (Spearman’s ρ = 0.15, *p* = 0.633; ρ = −0.01, *p* = 0.983; and ρ = −0.14, *p* = 0.736, respectively). Owing to higher data completeness, the 6-month timepoint was selected for a scatter-plot illustration (Fig. 4). Data points are widely dispersed across the full range of %RWG and %TWL, without evidence of a linear or non-linear relationship. Patients with both low and high %RWG demonstrated highly variable postoperative weight loss, ranging from minimal response to > 20%, with no consistent trend across the spectrum. These exploratory findings suggest that pre-revisional weight recurrence alone may be insufficient to predict postoperative weight loss following revisional surgery.

## Discussion

In this retrospective cohort, we compared pouch revisional surgery and Minimizer ring implantation as strategies to address recurrent weight gain or functional failure after RYGB. Both approaches resulted in postoperative weight loss, with no statistically significant difference during short- and mid-term follow-up. However, their safety and tolerance profiles differed within the available follow-up period. Previous studies have highlighted a recurring trade-off between durability and technical simplicity in revisional bariatric surgery. The primary objective of this study was to evaluate not only weight loss outcomes, but also the cumulative burden of complications and re-interventions associated with each revisional strategy. These findings must be interpreted in light of the study’s retrospective design, small cohort size, and limited follow-up. Particularly the limited adequacy of follow-up data beyond 6 months, in the context of early time to ring removal after 8.36 months may restrict generalizability and preclude definitive conclusions regarding late functional outcomes. However, this variability is clinically relevant, as the same revisional concept can yield divergent durability and complication profiles depending on the underlying failure mechanism, procedural complexity, and patient selection. This reinforces that revisional bariatric surgery represents a spectrum of risk rather than a uniform category [6]. Concordant radiographic and endoscopic findings of a dilated pouch and wide gastrojejunal anastomosis are viewed as a determinant of RWG after RYGB. However, weight recurrence after RYGB is multifactorial and cannot be explained by anatomy alone. Behavioral, nutritional, psychological, hormonal and metabolic factors may also influence outcomes. Anatomical correction should therefore be considered one component of multidisciplinary revisional decision making and follow-up. In this context ring implantation has frequently been proposed as a less complex alternative to anatomical revision [2, 7]. In our small cohort, this proposed technical simplicity did not translate into superior durability. Although a pouch revision required longer operative times, it directly addressed anatomical failure and was associated with a low rate of late complications and no need for reintervention beyond the index hospitalization. The Minimizer ring group carried a higher burden of late intolerance and device-related complications. Nearly half of implanted rings (42.9%) required removal due to dysphagia, intolerance, or severe adverse events. This included one case of ring dislocation with intestinal ischemia. Previous studies have reported that revisional ring placement, particularly after RYGB, carries a high likelihood of intolerance and re-intervention [4, 8, 9]. These findings closely align with our experience. Although no statistically significant difference in early postoperative complication rates was detected between groups, outcomes diverged, driven by the cumulative need for secondary interventions in the ring group. This observation highlights that short-term surgical safety alone may not adequately capture the clinical burden of revisional strategies. An additional finding of this study was the absence of any association between pre-revisional weight regain and postoperative weight loss. Correlation analysis demonstrated no significant relationship between RWG after RYGB and TWL after selected revisional procedures, suggesting that the magnitude of prior weight recurrence does not predict response to revisional intervention. Given the small sample size and limited statistical power, this finding does not exclude a predictive role of weight recurrence, but suggests that weight recurrence alone may be insufficient to guide revisional strategy selection.

### Efficacy

Both analyzed revisional strategies resulted in progressive weight loss over time, with no statistically significant differences in TWL at 3, 6 and 12 months. However, long-term data have demonstrated superior five-year excess weight loss and reduced weight regain in banded compared with non-banded RYGB [2]. Similar findings have been reported in large propensity score-matched cohorts, showing higher %TWL and a significantly lower risk of recurrent weight gain following ring-augmented RYGB [7]. These results support the potential efficacy of ring augmentation in appropriately selected patients but not necessarily for the revisional context of our cohort. Additional series combining ring placement with pouch resizing have also reported meaningful weight loss [9, 10]. Ring-augmented revisional approaches have demonstrated additional postoperative weight loss. Yet, device-related complications such as dysphagia and slippage may require reintervention or removal, potentially limiting long-term effectiveness [5]. This suggest favorable and durable weight loss outcomes for ring augmentation in the primary RYGB setting. However, their applicability to revisional cohorts remains limited as prior surgical alterations, and indication heterogeneity may influence postoperative effectiveness and tolerability. In this context also pharmacological strategies can achieve clinically meaningful reversal of weight regain after RYGB, with pharmacological approaches offering a less invasive alternative in selected patients. These findings, however, are not directly relevant to the present analysis [10]. In our cohort we did not observe a significant weight loss advantage for the Minimizer ring group within the first 12 months. This discrepancy likely reflects differences in indication, patient selection, and the revisional nature of our cohort, as well as the high rate of early ring removal due to intolerance. Consistent with prior observations, the indication window for revisional ring augmentation appears narrow. The potential efficacy of ring implantation may be substantially reduced when device-related complications limit sustainable retention [11]. However, the mechanisms of symptom control, particularly regarding dumping remission, may diverge over time. A well-tolerated ring provides fixed restriction and can support sustained symptom control. In contrast, pouch reconfiguration may be susceptible to secondary dilation and recurrent dumping over time. Therefore, we acknowledge potential differences in efficacy between these revisional strategies. Postoperative behavior, dietary adherence, and multidisciplinary follow-up may have influenced outcomes, but these factors were not systematically assessed. The perceived treatment intensity of pouch revision may also have affected patient engagement and postoperative management. Since between-group differences were not significant, this interpretation remains speculative and hypothesis-generating.

### Safety and complications

Early postoperative complication rates did not differ significantly between pouch revision and Minimizer ring implantation. In the pouch revision group, one major complication required reoperation, and one patient experienced transient dysphagia managed conservatively. No patient in this group required further surgical or endoscopic intervention during follow-up. Published data indicates that pouch revisional surgery after RYGB is associated with a measurable but variable early morbidity, reflecting the technical complexity of revisional surgery [12–14]. Some report variable but relevant early morbidity after pouch revisional surgery, with complication rates ranging from approximately 15% in homogeneous laparoscopic pouch-resizing cohorts to nearly 30% in more heterogeneous revisional populations. Major complications (Clavien–Dindo ≥ III) can occur in 10–11% of cases, particularly in resection procedures performed in anatomically altered fields, while reoperation rates remain relatively low when revision is limited to targeted pouch correction [12–14]. Across pouch-based revisional strategies, including pouch resizing and reshaping, anatomical correction is typically applied as a late-step intervention. These approaches achieve midterm stabilization around the prior nadir. Perioperative morbidity is clinically relevant but generally acceptable, with reported rates of approximately 12–27% for minor and 10–15% for major complications [15, 16]. Our pouch revision cohort demonstrated a low rate of major complications, suggesting that pouch revision can be performed with acceptable perioperative safety in selected patients. In the Minimizer ring group, however, late morbidity was substantial, with > 40% of rings requiring removal. This resulted in a higher cumulative reintervention burden despite similar early postoperative outcomes. While a subset of patients benefits from restored restriction, poor tolerance can rapidly convert the device into a liability requiring removal. Across revisional ring-based cohorts, late functional intolerance repeatedly emerges as the dominant driver of secondary interventions, underscoring that technical simplicity can translate into downstream clinical complexity [17]. Reintervention rates of up to 65% following revisional adjustable gastric banding after RYGB, largely due to dysphagia and reflux have been described [4]. Dysphagia and food intolerance emerge as key drivers for ring removal, with reported removal rates ranging from 12% to 25% at mid- to long-term follow-up [9, 18]. Rare but severe complications such as ring migration, bowel obstruction and ischemia have also been described, consistent with the serious adverse event observed in our ring cohort [8, 14]. Taken together, pouch revision entails early surgical risk, whereas ring implantation may shift morbidity toward delayed intolerance and reintervention. Endoscopic and surgical management of ring-related complications often requires repeated interventions, further contributing to cumulative morbidity [14]. By avoiding foreign body related complications, pouch revision may reduce cumulative reinterventions and offer a more durable revisional strategy.

### Functional outcomes and strategic implications

Functional tolerance differed between groups despite no statistically significant difference being detected in weight loss outcomes. In our cohort, Minimizer ring implantation was more frequently associated with restrictive intolerance, including dysphagia, vomiting, and food aversion, often leading to ring removal. This is consistent with prior studies reporting inferior food tolerance, reduced dietary variety, and lower nutritional satisfaction in ringed patients [8]. Altered dietary patterns, including higher fat intake and reduced protein and fiber consumption, further suggest adaptation to intolerance rather than optimized nutrition [3]. These effects appear to be related to the degree of mechanical restriction, with smaller ring diameters associated with increased dysphagia and reduced food satisfaction [3, 5, 8, 14, 18]. In contrast, pouch revision appeared to preserve better food tolerance, which may be advantageous when quality of life is prioritized. Considering efficacy, safety, and quality of life, revisional strategy selection after RYGB should prioritize functional outcomes over technical simplicity. Although Minimizer ring implantation offers a straightforward method to restore restriction, its effectiveness in the revisional setting appears highly dependent on patient tolerance and device retention [4, 17, 18]. When intolerance or device related complications limit retention, efficacy in revisional cohorts may converge with that of purely anatomical pouch correction [5]. By contrast, pouch based revisions directly address structural failure and concentrate surgical risk in the perioperative period, offering a more predictable postoperative course [15, 16]. These approaches differ not only in technique but also in the timing and mechanism of risk. Ring implantation shifts morbidity toward delayed functional intolerance and reintervention, whereas pouch revision concentrates risk early while avoiding foreign body related failure modes [4, 17]. Recognizing this distinction supports a tailor-made approach in which anatomical findings, symptoms, and tolerance for potential re-intervention guide strategy selection. This is consistent with our exploratory finding that pre-revisional weight recurrence alone may be insufficient to predict postoperative outcome. It reinforces that indication should be guided by mechanism of failure rather than extent of weight recurrence.

## Limitations

This study has several limitations. The small sample size and retrospective single-center design limit statistical power and generalizability. Selection bias and residual confounding cannot be excluded. Follow-up was limited, and data completeness was further affected by the high rate of Minimizer ring removal, restricting the mid-term assessment of and potentially biasing group comparisons. Finally, heterogeneity in patient compliance, surgical indications, baseline weight recurrence, and pooled pouch-revisional procedures may have influenced outcomes and limited direct comparability between groups.

## Conclusion

In this retrospective single-centre cohort, both pouch revision and Minimizer ring implantation resulted in postoperative weight loss, with no significant between-group difference during short- to mid-term follow-up. However, ring implantation was associated with more intolerance and secondary interventions, including ring removal. While ring implantation offers technical simplicity and durable restriction when tolerated, it was associated with a high rate of late intolerance and device-related complications in the revisional setting, frequently necessitating removal. In contrast, pouch revision directly addressed anatomical failure and demonstrated a more predictable postoperative course, with fewer short-term complications and preserved functional tolerance. Given the small sample size, retrospective design, limited follow-up, and potential selection bias, these findings should be considered hypothesis-generating. Nevertheless, these findings suggest that in selected revisional patients with recurrent weight gain or functional failure, anatomical correction without foreign body implantation may offer a safe and patient-centered alternative. Surgical decision making should therefore consider safety, symptom control, quality of life, and reintervention burden alongside procedural simplicity.

## Data Availability

The datasets generated and/or analyzed during the current study are not publicly available due to patient confidentiality and institutional restrictions but are available from the corresponding author upon reasonable request.

## Abbreviations

BAROS: Bariatric Analysis and Reporting Outcome System
BMI: Body mass index
EWL: Excess weight loss
IQR: Interquartile range
RYGB: Roux-en-Y gastric bypass
RWG: Recurrent weight gain
STROBE: Strengthening the Reporting of Observational Studies in Epidemiology
TWL: Total weight loss

## References

[1] Stubbs RS (2014) What makes a gastric bypass a good gastric bypass? Opinion and hypothesis. WJSP 4(2):48. 10.5412/wjsp.v4.i2.48

[2] Lemmens L (2017) Banded Gastric Bypass: Better Long-Term Results? A Cohort Study with Minimum 5-Year Follow-Up. OBES SURG April 27(4):864–872. 10.1007/s11695-016-2397-4 10.1007/s11695-016-2397-4 PMC5339319 27714527

[3] Faria SL, Faria OP, Cardeal MDA (2014) Comparison of weight loss, food consumption and frequency of vomiting among Roux-en-Y gastric bypass patients with or without constriction ring. ABCD arq bras cir dig 27(suppl 1):43–46. 10.1590/s0102-6720201400s100011 25409965 10.1590/S0102-6720201400S100011 PMC4743518

[4] Lazaridis II, Kraljević M, Süsstrunk J, Köstler T, Zingg U, Delko T (2021) Revisional Adjustable Gastric Band in Roux-en-Y Gastric Bypass—Is It Worth It? J Gastrointest Surg Dezember 25(12):3056–3063. 10.1007/s11605-021-05045-7 10.1007/s11605-021-05045-7 PMC8654708 34100249

[5] Van Helmond SC, Jense MTF, Verkoulen GHJM, De Witte E, Broos PPHL, Greve JWM (2025) u. a. Pouch Revision in Combination with Placement of a MiniMizer Ring as a Revisional Procedure in Patient with Suboptimal Clinical Response or Recurrent Weight Gain After RYGB. OBES SURG August 35(8):2990–2997. 10.1007/s11695-025-07984-5 10.1007/s11695-025-07984-5 PMC12380964 40616624

[6] Tran DD, Nwokeabia ID, Purnell S, Zafar SN, Ortega G, Hughes K (2016) u. a. Revision of Roux-En-Y Gastric Bypass for Weight Regain: a Systematic Review of Techniques and Outcomes. OBES SURG Juli 26(7):1627–1634. 10.1007/s11695-016-2201-5 10.1007/s11695-016-2201-5 27138603

[7] Jense MTF, Bruinsma FFE, Nienhuijs SW, Liem RSL, De Mheen PJM (2025) Greve JWM, u. a. Ring Augmentation of the Roux-en-Y Gastric Bypass: A Propensity Score Matched Analysis of 5-Year Follow-Up Results. OBES SURG März 35(3):884–893. 10.1007/s11695-025-07706-x 10.1007/s11695-025-07706-x PMC11906517 39883395

[8] Szymczak P, Grzybowska ME, Grzeczka A, Szymański M, Wydra DG (2024) MiniMizer Gastric Ring displacement at 31 weeks of gestation as a life-threatening complication. OBES SURG November 34(11):4263–4266. 10.1007/s11695-024-07521-w 10.1007/s11695-024-07521-w PMC11541374 39367262

[9] Franca FT, De Almeida Gentile JK, Migliore R, Bueno De Souza PMS, Assef JC (2021) Evaluation of weight loss and quality of life in morbidly obese patients undergoing y de roux gastric bypass surgery with reduction ring and without ring, after the first year of follow-up. Gastroint Hepatol Dig Dis 4(1). 10.33425/2639-9334.1058

[10] Horber FF, Steffen R (2021) Reversal of Long-Term Weight Regain After Roux-en-Y Gastric Bypass Using Liraglutide or Surgical Revision. A Prospective Study. OBES SURG Januar 31(1):93–100. 10.1007/s11695-020-04856-y 10.1007/s11695-020-04856-y PMC7808975 32691401

[11] Van Dam KAM, De Witte E, Broos PPHL, Greve JWM, Boerma EJG (2024) Short-term safety and effectiveness of conversion from sleeve gastrectomy to Ring augmented Roux-en-Y gastric bypass. BMC Surg 19 September 24(1):266. 10.1186/s12893-024-02552-7 10.1186/s12893-024-02552-7 PMC11411827 39300438

[12] Al-Bader I, Khoursheed M, Al Sharaf K, Mouzannar DA, Ashraf A, Fingerhut A (2015) Revisional Laparoscopic Gastric Pouch Resizing for Inadequate Weight Loss After Roux-en-Y Gastric Bypass. OBES SURG Juli 25(7):1103–1108. 10.1007/s11695-015-1579-9 10.1007/s11695-015-1579-9 PMC4460267 25599857

[13] Fronza JS, Prystowsky JB, Hungness ES, Nagle AP (2010) Revisional bariatric surgery at a single institution. Am J Surg November 200(5):651–654. 10.1016/j.amjsurg.2010.07.012 10.1016/j.amjsurg.2010.07.012 21056147

[14] Campos JM, Evangelista LF, Ferraz ÁAB, Galvao Neto MP, De Moura EGH, Sakai P (2010) u. a. Treatment of ring slippage after gastric bypass: long-term results after endoscopic dilation with an achalasia balloon (with videos). Gastrointest Endoscopy Juli 72(1):44–49. 10.1016/j.gie.2010.01.057 10.1016/j.gie.2010.01.057 20493480

[15] Hehl SJ, Birrer DL, Hauser R, Gero D, Thalheimer A, Bueter M (2024) Gastric pouch resizing for recurrent weight gain after Roux-en-Y gastric bypass—does it have its rational? Obes Surg 34(12):4369–4377. 10.1007/s11695-024-07581-y 39531140 10.1007/s11695-024-07581-y PMC11671430

[16] Borbély Y, Winkler C, Kröll D, Nett P (2017) Pouch Reshaping for Significant Weight Regain after Roux-en-Y Gastric Bypass. OBES SURG Februar 27(2):439–444. 10.1007/s11695-016-2329-3 10.1007/s11695-016-2329-3 27510586

[17] Boerboom A, Aarts E, Lange V, Plamper A, Rheinwalt K, Linke K (2020) u. a. Banding the Pouch with a Non-adjustable Ring as Revisional Procedure in Patients with Insufficient Results After Roux-en-Y Gastric Bypass: Short-term Outcomes of a Multicenter Cohort Study. OBES SURG März 30(3):797–803. 10.1007/s11695-019-04361-x 10.1007/s11695-019-04361-x 31898043

[18] Okkema S, Boerboom A, Den Hengst W, Aarts E, Berends F, Hazebroek E (2025) Five-year outcomes of a randomized controlled trial evaluating a non-adjustable ring in Roux-en-Y gastric bypass. Surg Endosc April 39(4):2324–2334. 10.1007/s00464-025-11545-3 10.1007/s00464-025-11545-3 PMC11933145 39953277

